# The Role of Cannabinoidergic System in Prenatal Neurodevelopment

**Published:** 2013

**Authors:** Nima Naderi

The cannabinoidergic system acquired a reputation as the most abundant G-protein coupled receptor in the CNS that acts as retrograde modulator of neurotransmitter release. Recently, endocannabinoids (ECBs) have been highlighted as neurodevelopmental signaling cues that exert a regulatory role in brain development. The interruption in ECB system elements (including receptors and enzymes of synthesis and degradation) in the developing brain is in relation with postnatal CNS pathophysiology. The rapid rates of ECB synthesis/degradation reveal the existence of a dynamically regulated ECB tone during the active neurogenesis. The cannabinoid CB1 receptor is expressed from very early stages of embryonic development, even before the appearance of the neural tube and ectoderm development. CB1 is present in trophoblast stem cells and its deletion results in reduced cell proliferation and differentiation that is followed by aberrant formation of placenta and compromised embryo implantation. In addition to CB1 receptor expression, the other cannabinoid receptor, the CB2 receptor, is also present in the inner cell mass, and is involved in embryonic stem-derived hematopoietic cell proliferation and differentiation. In mammals, CB1 receptor expression is characterized by its abundant levels in white matter areas (where the axons of neural cells are present), with their levels progressively increasing from prenatal stages to adulthood in grey matter areas (where mostly occupied by neural cell bodies and dendrites) during neural development. The expression of CB1 receptor during the development occurs in active neurogenesis and axonal migration and prior to the synaptic maturation and neuronal activity. Therefore, the action of CB1 receptors on neurodevelopment is likely to be independent of their regulatory role of neurotransmitter release and neuronal activity. At embryonic stage and during cortical development, CB1 receptors are present in pioneer neurons which develop cortical plate. At these stages, the CB1 receptor is also present in the hippocampus. Later, CB1 receptors are heterogeneously distributed through cortical layers and the hippocampus, in both excitatory glutamatergic projection neurons and GABAergic interneurons. In human fetal brain, there is growing evidence of CB1 receptor expression throughout the cerebral cortex, hippocampus, caudate nucleus, putamen and cerebellum. High densities of CB1 receptors are detected during the prenatal development in brain areas that are practically devoid of these receptors later in the adult brain. The early expression and function of the cannabinoid receptors during CNS development and its transient localization in prenatal stages suggest a specific role of the ECB system in human brain development, with potential implications in neuropsychiatric disorders. The two putative ECBs, anandamide (AEA) and 2-arachidonoylglycerol (2AG) can act in an autocrine or paracrine manner on neural progenitors. The ECB system is initially involved in axonal growth but is later redistributed to dendrites where it plays the retrograde modulatory role in neurotransmission. During the cortical and retinal development, ECB production participates in axon guidance and neurite outgrowth. The regulatory role of the CB1 receptor in neuronal generation and maturation in the embryonic brain is preserved in the neurogenic niches of the adult brain. Neural progenitors in adult neurogenic brain areas also express the CB1 receptor and produce ECB ligands. CB1 receptors are expressed in neural progenitor cells of the subgranular and subventricular zones, in which they drive progenitor proliferation and neural differentiation in adult brain. Therefore, the neurodevelopmental role of the ECBs is continued in the mature nervous system. Altered cannabinoid signaling (*i.e*. either hyper- or hypo-function of the CB1 receptor) can exert long-lasting consequences in adult brain neuronal function by modifying the actively developing brain. Neurodevelopmental disorders can be originated by subtle or severe alterations of various neurogenic processes, including neuronal generation, migration, maturation and connectivity that are responsible for adult brain dysfunction. Among developmental disorders, cortical alterations constitute an important example of how embryonic deficits affect adult neurological function. Genetic polymorphisms of cannabinoid receptors can induce subtle changes during the development by influencing signaling strength or duration and later, when synaptic transmission ensues, by influencing the appropriate balance of neuronal activity. Likewise, mutations of ECB-metabolizing enzymes, including degrading or synthesizing enzymes, may result in changes in enzymes activity that would increase or reduce the ECB tone. In this regard, ECB degrading enzyme polymorphisms have been associated with drug abuse behaviors, neurodegenerative diseases, hearing loss, retinitis pigmentosa and cataract, and finally demyelination and cerebellar ataxia. CB1 receptor signaling can be influenced as well by prenatal exposure to marijuana-derived cannabinoids or by contact with drugs targeting either directly or indirectly the ECB system. The neurobiological consequences of plant-derived cannabinoid intake on the pre- and postnatal stages have been recently reviewed and indicate that the brain development period is of especial susceptibility. Interference on CB1 receptor signaling may affect different aspects of neural cell development, including neuronal generation and specification (embryonic stages), glial development (postnatal stages) and neuronal maturation and connectivity. In addition, CB1 receptor expression, first in white matter and later in postnatal grey matter, participates in the appropriate integration of sensory information input. Polymorphisms of both the CB1 and the CB2 encoding genes may reduce or enhance G-protein mediated signaling and have been associated to the major depression, psychoses and schizophrenia. The glutamatergic neuronal dysfunction hypothesis of schizophrenia suggests that malfunction of the CB1 receptors in pyramidal neurogenesis may contribute to the pathogenesis of psychoses or schizophrenia symptoms. It should be noted that although malfunction of the ECB system may be one of the causes underlying neuronal dysfunction, alternatively, the CB1 receptor and ECB-metabolizing enzymes are also able to adapt to the aberrant neuronal homeostasis as an attempt to counteract the changes of neuronal transmission. In this regard, cortical glutamic acid decarboxylase deficiency, a typical neurochemical marker of schizophrenia, results in lower CB1 receptor expression. It remains unknown whether these kind of ECB system adaptations exert positive effects to cope with those alterations, or worsen the pathological processes.

The other consequence of cortical development alterations is the appearance of epileptic foci due to the alterations in neuronal excitability. Considering the dual role of the CB1 receptor in the generation and maturation of excitatory and inhibitory neurons, it can be predicted that CB1 receptor-dependent signaling alterations would impact the appropriate excitation/inhibition balance of the mature brain during the development. Thus, unbalanced CB1 receptor activity and its consequences in cortical pyramidal neurogenesis may elicit the epileptic syndromes. In addition to the excitatory neuronal alterations, unbalanced generation of interneuron populations may also contribute to the developmental epilepsies. Disruption of cortical GABAergic interneuron development could exert GABAergic cell type-specific deficits, epilepsy and behavioral dysfunction. Retrograde ECB messengers are key regulators of synaptic plasticity, both of inhibitory synapses (depolarization-induced suppression of inhibition and long-term depression of inhibition) and excitatory synapses (depolarization-induced suppression of excitation and long-term depression of excitation). It is important to note that, as within the early stages of brain development, GABA is excitatory instead of inhibitory. CB1 receptor activation and subsequent inhibition of GABA release would result in different outcomes depending on the developmental stage in which the ECB system function is altered.

In Conclusion, the cannabinoid receptor signaling is contributed to appropriate nervous system formation. At early developmental stages, a precisely regulated ECB function act as signaling cues in neurogenic niches. CB1 receptor activity exerts a critical regulatory role in different neural cell process, including cell proliferation, neuronal specification, and neuronal migration and morphogenesis. The ECB system exerts a key regulatory role in cortical developmental and this may have significant consequences in adult brain function, including the maintenance of an appropriate balance of neuronal excitation/inhibition activity and the susceptibility to suffer epilepsy or neuropsychiatric disorders.

*Nima Naderi is currently working as an Assistant Professor at the Neuroscience Sciences Research Center and Department of Pharmacology and Toxicology, School of Pharmacy, Shahid Beheshti University of Medical Sciences, Tehran, Iran. He could be reached at the following E-mail address: naderi@sbmu.ac.ir*

